# Review of progress and challenges of key mechanical issues in high-field superconducting magnets

**DOI:** 10.1093/nsr/nwad001

**Published:** 2023-01-06

**Authors:** You-He Zhou, Dongkeun Park, Yukikazu Iwasa

**Affiliations:** Key Laboratory of Mechanics on Disaster and Environment in Western China attached to the Ministry of Education of China, Lanzhou University, Lanzhou 730000, China; Department of Mechanics and Engineering Sciences, College of Civil Engineering and Mechanics, Lanzhou University, Lanzhou 730000, China; The Plasma Science and Fusion Center, Francis Bitter Magnet Laboratory, Massachusetts Institute of Technology, Cambridge, MA 02139, USA; The Plasma Science and Fusion Center, Francis Bitter Magnet Laboratory, Massachusetts Institute of Technology, Cambridge, MA 02139, USA

**Keywords:** REBCO second-generation high-temperature superconducting coated conductors (REBCO 2G-HTS CCs), high-field magnets, mechanics, screening-current effect

## Abstract

The development of modern science and technology requires high magnetic fields exceeding 25T. Second-generation high-temperature superconducting wires, i.e. REBCO (REBa_2_Cu_3_O_7_-x, RE refers to Y, Gd, Dy, Eu and other rare-earth elements) coated conductors (CCs), have become the first choice for high-field magnet construction because of their high irreversible magnetic field. The mechanical stresses caused by manufacturing, thermal mismatch and Lorenz forces closely influence electromagnetic performance during operation for REBCO CCs. In addition, the recently studied screen currents have effects on the mechanical characteristics of high-field REBCO magnets. In this review, the experimental and main theoretical works on critical current degradation, delamination and fatigue, and shear investigations on REBCO CCs, are reviewed at first. Then, research progress on the screening-current effect in the development of high-field superconducting magnets is introduced. Finally, the key mechanical problems facing the future development of high-field magnets based on REBCO CCs are prospected.

## Teaser

In this review, they summarized the research progress of superconductor mechanics on REBCO coated conductors and high-field magnets, and presented the key challenges on the future development of superconductor mechanics.

## INTRODUCTION

**Figure 1.**
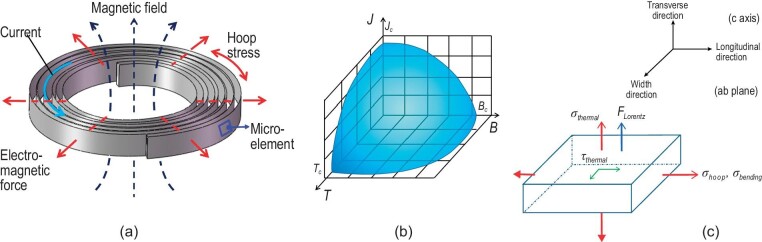
(a) Schematic of a single-pancake coil in the magnet, reproduced from ref. [14]. (b) The critical temperature, critical magnetic field and critical current density of a superconductor. (c) Stress applied in the microelement body of the CC.

High-field superconducting magnets have potential application prospects in future accelerators, nuclear magnetic resonance (NMR), magnetic resonance imaging (MRI), fusion reactors and other large-scale advanced devices. At present, superconducting magnets can be divided into two categories. One is low-temperature superconducting (LTS) magnets made of NbTi and/or Nb_3_Sn materials. They have already been commercialized (in MRI, NMR, etc.), and successfully demonstrated in accelerators and so on [1–3]. The other is superconducting magnets based on high-temperature superconducting materials. Compared with Bismuth-based superconductors and MgB_2_ high-temperature superconducting (HTS) materials, second generation REBCO (REBa_2_Cu_3_O_7_-x, RE refers to Y, Gd, Dy, Eu and other rare-earth elements) HTS coated conductors (CCs) have higher mechanical strength and critical current. In particular, its excellent current-carrying capacity (450–500 A at 4.2 K and 19 T [4]) under high magnetic field makes it suitable for high-field superconducting magnets over 15 T. In addition, REBCO CCs have been commercialized on a large scale. Now REBCO CCs are produced worldwide by many companies from the USA [5,6], Japan [7] and China [8] etc. One type of magnet consists of pancake winds with flat REBCO CCs, shown in Fig. 1(a). The superconducting magnet properties are constrained by the non-linear electromagnetic constitutive relation formed by the interaction between critical temperature, critical magnetic field and critical current density, as shown in Fig. 1(b). It is well known that the magnetothermal instability of a superconducting material is closely related to the safe and stable operation of the superconducting device. Jing *et al*. developed a series of numerical models to study the flux avalanches and mechanical failure of superconductors [9–13]. The numerical results were in good agreement with experiments, and revealed some new findings about the thermal-magnetic-mechanical instability behavior of the superconductors. REBCO CCs are inevitably subjected to complicated mechanical loadings, such as the assembly stress, thermal stress and electromagnetic stress. Any microelement in a superconducting magnet is subjected to the superposition of normal stress and tangential stress, as displayed in Fig. 1(c).

As early as 1976, Ekin discovered that the critical current of Nb_3_Sn would degrade non-linearly with an increase in strain [15]. Later, a large number of experimental results showed that REBCO superconducting materials also show a similar phenomenon. Therefore, the mutual coupling between strain and current-carrying characteristics was inevitable in the application of REBCO CCs. For the convenience of engineering design, researchers defined the concept of irreversible strain under uniaxial tensile load to characterize the degradation behavior of critical current with strain. Once the deformation of the superconducting tape exceeds the critical strain, the critical current degenerates irreversibly, which indicates that part of the superconducting material may be damaged. In addition, because the REBCO CC is a laminated structure having a potential risk of lateral delamination, it is also difficult to figure out how to accurately measure the lateral allowable stress, which can guide the engineering design. Therefore, in the first part of this paper, research progress on the axial tension and lateral delamination strength of the REBCO CC (YBCO (YBa_2_Cu_3_O_7_-x) CC is often used as an example) is reviewed.

As is known, with an increase in the magnetic field strength the screening-current effect in a magnet becomes more and more important. Traditional magnet design methods often ignore the relationship between mechanical characteristics and the screening-current effect. They only regard the mechanical responses as direct outputs without considering coupling mechanical effects. Previous studies have shown that this would often lead to it being difficult for the design index of the magnetic field of superconducting magnets to reach expectations. In the second part of this paper, the research progress on mechanical characteristics and the screening-current effect in superconducting magnets is summarized. Certainly, there are many comprehensive articles on the development of superconducting magnets based on high-temperature superconducting materials [16–18]. These articles summarize the current research status of superconducting magnets and the difficulties and challenges from different views. In this review, the key mechanical problems facing the development of high-field magnets based on REBCO CCs are summarized. And more attention should be paid to mechanical effects in the development process of future high-field magnets.

## ELECTROMECHANICAL PROPERTIES OF REBCO CCs

**Figure 2.**
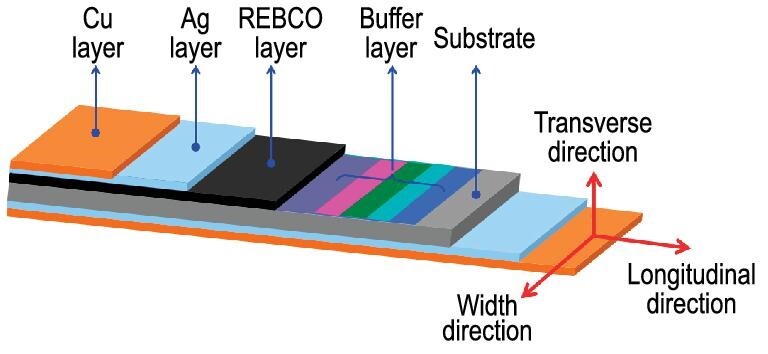
Structure of the layered structure of the REBCO 2G HTS CC, in which RE refers to Y, Gd, Dy, etc.

Compared to LTS wires such as Nb_3_Sn and NbTi, the critical temperature and magnetic field of HTS wires, e.g. the Bi-based first-generation (1G) HTS wires and the REBCO CCs, are far beyond those LTS materials, making it possible to construct magnets exceeding 25 T. In contrast with 1G HTS wires, REBCO CCs have larger tensile mechanical strength and large critical current density at 77 K. As a result, REBCO CCs emerged as a potential candidate for high-field magnets and power applications [19,20]. Through years of effort, several physical and chemical deposition techniques have been developed, giving CCs layered structures that are significantly improved, with a long length (>1 km) and high performance (230–305 A at 4.2 K and 18 T [4]). The architecture of REBCO CCs is displayed in Fig. 2.

Prototypes of transmission cables, motors and magnets are manufactured with REBCO CCs [21–23], and a huge market with great potential and wide prospects is coming. Despite the superior merits of REBCO CCs, the working conditions of this material are very extreme and always involve cryogenic temperatures of 4.2–77 K and magnetic fields up to tens of Tesla, hence the large thermal stress and electromagnetic forces (EMFs) that are exerted on these CCs, as shown in Fig. 1. The reported hoop stress can be as large as hundreds of MPa [24,25], and the thermal-stress-induced delamination behavior can be a serious problem in an epoxy-impregnated magnet coil [26,27]. The damage caused by a huge electromagnetic force has been a constraint for achieving higher magnetic fields [28,29]. As a ‘practical’ magnet-grade conductor, REBCO CCs pursue not only the high critical current density *J_c_*, but also the ability to withstand high mechanical stress without major deterioration of the transport properties when bent or stressed during handling, winding or operation under extreme environments [30]. In this part, we review the electromechanical property investigation of REBCO CCs from aspects of experimental, theoretical and numerical analyses. The remaining challenges with regard to experimentation and mechanism analysis are also reported, aiming to promote the future improvement of REBCO CCs with high performance for scaled-up applications.

### The electromechanical property of REBCO CCs in a longitudinal direction

#### Experimental approaches for longitudinal electromechanical investigation

**Figure 3.**
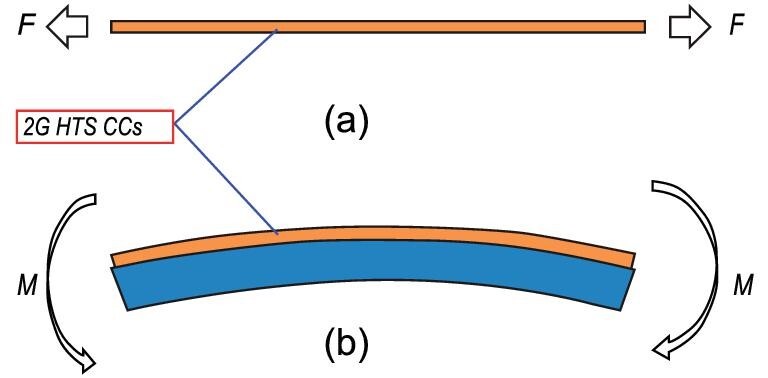
Schematic of two types of longitudinal strain loading. (a) Direct stretching by tensile machine. (b) Bending through the attached loading module.

To investigate the longitudinal strain influence on the critical current *I_c_* in REBCO CCs, the strains are exerted on samples through two methods: one is direct stretching, and the other is bending through the attached loading module, shown in Fig. 3.

In the stretching loading types, the tensile force/strain can be recorded from the load cell and extensometer, respectively. In the direct stretching method, clamps serve the dual purpose of electrical contacts and grips for applying strain. The *I_c_* is determined by a four-point testing method. Stress $\sigma $ vs. strain $\varepsilon $ curve and $\varepsilon $ dependence of *I_c_* curve can be obtained. However, sometimes non-uniform strain distribution near the clamps causes pre-quench during the test. Direct stretching methods are utilized in research groups in Lanzhou University [31,32], Kyoto University [33], Andong National University [34] and Twente University [35]. In the bending approach, the CC sample is soldered onto a metal beam, such as U-spring, Pacman, Walters spring. Tensile or compressive strains can be applied to the CC samples by changing the bending diameters through the bending moments applied to the beam, and can be expressed as $\varepsilon = {y / R}$, where *y* and *R* are the distance from a neutral axis to the REBCO film, and bending radius. Since no clamps are used, the non-uniform stress distribution is avoided. Compared with direct stretching, bending can provide more uniform tensile and compressive strains. The bending approaches are adopted among research groups in Twente University [36], Geneva University [37], Durham University [38] and Colorado University [39]. Both types of loading frames have been developed for 77 K self-field and magnetic-field tests at 77K [31,36,40], magnetic field tests at different temperatures [37–39,41,42]. For direct measurement of the internal strain of the superconducting layer in the composite YBCO CCs, the diffraction techniques of synchrotron radiation [43] or neutron diffraction [44] were used combined with a tensile module to monitor the Bragg peaks of the crystal plane. Recently, Liu *et al*. developed a loading frame combined with an magneto-optical (MO) imaging system to monitor the real-time magnetic flux evolution of the CC under tensile strain [32,45]. Zhou *et al.* constructed a loading facility with an incorporated racetrack superconducting magnet providing a magnetic field up to 3.5T parallel to the c-axis [46]. The loading frame was cooled by Gifford-Mcmahon (GM) cryocooler so that different *I_c_*-strain curves could be acquired at different temperatures. This facility contains a quartz window enabling the global strain measurement of YBCO CCs by digital-image-correlation (DIC) method. As can be found, tensile devices have been developed with multiple functions in order to further reveal physical properties during straining.

#### The electromechanical behaviors of REBCO CCs with strain below the irreversible strain limit

Experimental results in the 77 K self-field condition have shown that the longitudinal strain/stress can reversibly degrade the *I_c_* below a strain/stress limit, while beyond the limit, the degrading behavior is irreversible. The corresponding limits of strain and stress are denoted as irreversible strain limit ${\varepsilon }_{{\rm{irr}}}$ and irreversible stress limit ${\sigma }_{{\rm{irr}}}$ [47]. In the reversible strain range, the strain dependence of *I_c_* can be expressed with a simple power-law expression as [48,49]:


(1) \begin{eqnarray*} {I}_c( {{\varepsilon }_a} ) = {I}_{cm}( {1 - a{{| {{\varepsilon }_a - {\varepsilon }_p} |}}^b} ), \end{eqnarray*}

where the *ϵ_a_*and *ϵ_p_* are the applied microscopic strain and the peak strain (it is thought to be residual strain caused by thermal or lattice misfit) in the superconducting layer, *I_cm_* is the maximum critical current where the intrinsic strain *ϵ_0_*(${\varepsilon }_0\,\,{\rm{ = }}\,\,{\varepsilon }_a - {\varepsilon }_p$) is zero, *a* is the strain-sensitivity parameter and *b* is the fitting parameter exponent. Details of the approaches used to calculate the strain sensitivity can be found in Ekin's book [50]. Based on the 3D/2D mixed-dimensional modeling technique, Gao *et al.* adopted a mixed-dimensional elastoplastic finite element method (FEM) model and provided numerical analyses of YBCO CCs during fabrication, cooling and under tensile load [51]. In their model, the residual strains accumulated during the fabrication and cooling processes are calculated by a multi-step modeling method that emulates the manufacturing process, through which a phenomenological critical-current-strain model based on the Ekin power-law formula and the Weibull distribution function is combined with the mixed-dimensional conductor model to predict the strain dependence behavior of the critical currents in the reversible and irreversible degradation strain ranges. Yong *et al*. presented a model based on a modified Ginzburg-Landau function [52]. This model explains how the pre-strain markedly influences the wave function and degrades the critical current in the deformable superconductor. Van der Laan *et al.* found a similar critical-current degrading behavior, which was also found in YBCO films with grain boundaries of different angles [53]. It was explained that the maximum current is obtained when the applied strain offsets the compressive strain within the grain boundary channels. Based on these results, Yue *et al.* proposed a new model for current transporting in the [001]-tilt low-angle grain boundary based on the strain energy of dislocation [54], expressed as


**Figure 4.**
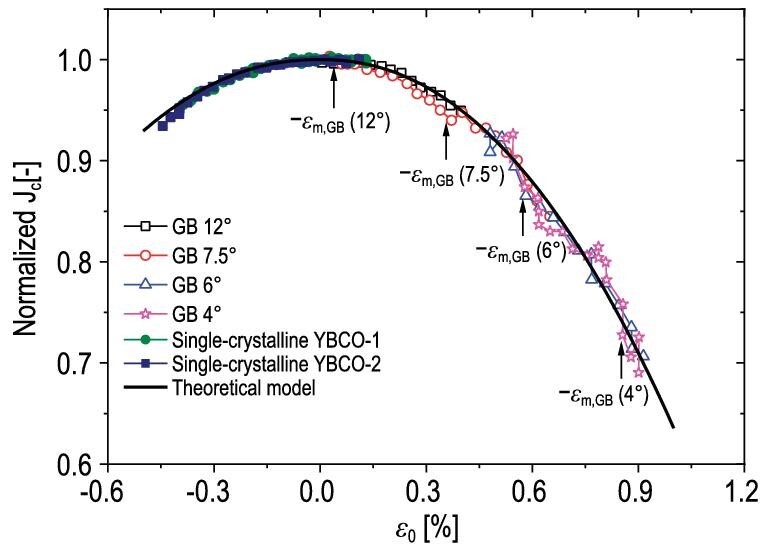
Experimental results versus the calculations from the model by Yue *et al*. [49].

(2) \begin{eqnarray*} {J}_{\varepsilon ,GB} &=& {J}_c\left( 0 \right)\exp \left( - \frac{\theta }{{{\theta }_c}}\right)\\ &&\left( {1 - \frac{{\alpha G{\rho }_0b_0^{2 + \Delta }}}{{\overline E \varepsilon _m^{2 + \Delta }}}\int\limits_{\omega }{{d\omega }}{{\left| {\varepsilon - {\varepsilon }_m} \right|}}^{2 + \Delta }} \right),\\ \end{eqnarray*}

where${J}_c( 0 )$is the critical current density when the sample does not experience strain, $\theta $ denotes the misorientation angle of the grain boundary, ${\theta }_{\rm{c}}$ is a constant (its value can be selected in the range of 3.2– 5°), ${\varepsilon }_m$denotes the maximum strain that existed within the superconducting channel when the applied strain is equal to zero, ${\rho }_0$ denotes the dislocation density without external strain, $\Delta $ is a positive parameter determined by the shape of the function, $\alpha Gb_0^{2 + \Delta }$ denotes the strain energy of single dislocation under free strain, and $\bar{E}$ is the average energy of the single dislocation within the grain boundary. Figure 4 displays the comparison between the experimental results and the presented model. One can see that a good agreement is obtained, and thus the clear physical meaning of the empirical parameters in the fitting formula has been confirmed.

In experiments with varying temperatures and magnetic fields, the strain dependence of the critical current becomes a little complex, and the main characteristics are summarized as follows:

- In an applied magnetic field parallel to the ab plane, the change of critical current density decreases with the low magnetic field and increases in the high magnetic field [55]. Where the magnetic field is applied along the c axis, the peak of the normalized *I_c_* emerges with the *ϵ_p_* shifted [33,39]. In a bending test, the normalized *I_c_*(*ϵ_a_*) shows two maxima under a magnetic field below 3 T, and both maxima are nearly the same and located at compressive and tensile sides. The relative magnitude of the peaks increases with magnetic field and reaches a maximum at ∼0.25 T. Both peaks disappear at an applied field of ∼3 T, and the reduction of *I_c_* with strain increases with magnetic field beyond 3 T [39].
- The strain dependence of the critical current in a self field or external field becomes insensitive when temperature is reduced [33,41,42]. In addition, the peak strain in a self field shifts to the compressive side as the temperature decreases [41] and cannot be determined just by thermo-strain [56] as previously suggested, because it is contrary to the thermo-strain analysis that the peak strain should be shifted to the tensile side.
- The existing models mentioned above hold well for the YBCO CCs at 77 K self field. The physical mechanisms of effects of temperature and magnetic field on *I_c_* (*ϵ_a_*) are still unclear. Therefore, a more comprehensive model still needs to be established so as to correctly predict the current behavior of YBCO CCs with varying temperatures and magnetic fields.

#### The cracking of the superconducting layer in REBCO CCs with strain beyond the irreversible strain limit

**Figure 5.**
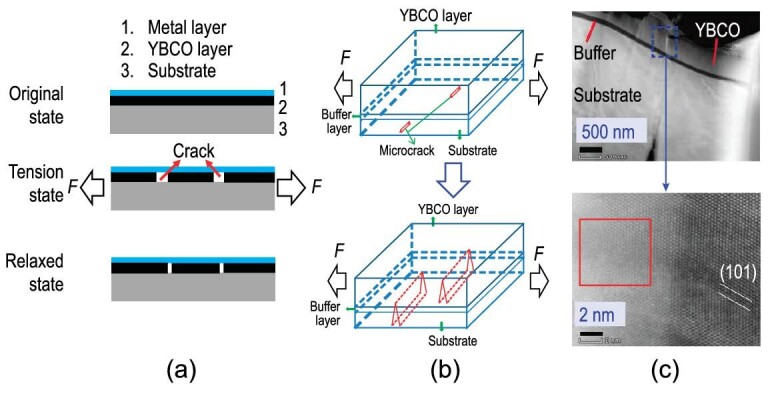
Fracture behavior of the superconducting layer in YBCO CCs. (a) Crack formation by discontinuous yielding of the substrate. (b) Crack extension from the substrate to superconducting layer. (c) Micro transmission electron microscope (TEM) results at the crack tip, where the amorphous phase exists and is indicated in the broken rectangle, reproduced from ref. [9].

When the applied strain in a second-generation (2G) HTS CC exceeds the *ϵ_irr_*, the reduction of critical current cannot be recovered after the stress is unloaded. Diffraction results have verified the ceramic superconducting layer fractures at *ϵ_irr_*, which are due to its brittle nature. The temperature and magnetic field do not affect the *ϵ_irr_*. The cracking behavior is thought to be related to discontinuous yielding [57], as shown in Fig. 5(a). A large localized strain initiates at the discontinuous yielding area, and therefore the misfit between the deformation of YBCO and substrate under the applied strain is mediated by the crack formation [58]. The results [14] from magnetic flux evolution under quasi-static loading reveal that the cracks are initiated from the substrate and extend along both tape thickness and width directions as shown in Fig. 5(b). The amorphous phases were found at the tip of the cracks, as displayed in Fig. 5(c).

#### Methods to improve the electromechanical property against the axial strain/stress

There are two ways of improving the *ϵ_irr_* of YBCO CCs, and the first way is to increase the stabilizing layer thickness or laminate a metal layer on YBCO CCs [34,59–64], where the increment of *ϵ_irr_* value ranges from 0.04% to 0.36%. The improvements are attributed to two reasons. One is that the additional metal layers have bigger coefficient of thermal expansion (CTEs) than YBCO, producing a larger compressive pre-strain in the YBCO layer during cool down and hence resulting in extra tensile strain to compensate. The other is that, under tensile stress, once the cracks initiate in the REBCO layer, they propagate in both thickness and width directions. These metal layers improve the toughness of the brittle ceramic layer because the added ductile phase shields (as a ligament) the crack initiation zones behind the crack tips, thus inhibiting and retarding crack propagation along the width direction. In spite of the increase of tolerant strain by an additional metallic layer, for materials like copper, with low yielding stress, an increase in the volume fraction of copper would lead to a decrease of ${\sigma }_{irr}$. Therefore, a trade-off between strength and protection should be considered in engineering design [62]. The second way is to solder YBCO CCs on a pre-stretched steel, so that a pre-compressive strain exists in the YBCO CCs. Thus, a larger tensile *ϵ_irr_* value is obtained by canceling the pre-compressive strain. This approach shows that the increment of the *ϵ_irr_* of the YBCO CCs is ∼0.34% [65].

#### Fatigue properties of REBCO CCs

For superconducting devices such as superconducting magnets, motors, transformers and magnetic energy storage devices, the coils inside, based on REBCO CCs, always involve situations of charge/discharge cycles, repeated thermal cycles and alternating current transportation. The resultant periodic electromagnetic force and thermal mismatch stress will apply on the CCs. As a result, the CCs will be subjected to alternating stress/strain. Thus, evaluating fatigue properties of REBCO CCs is important for their practical application. The fatigue investigations on REBCO CCs can be classified into two categories: one is high-cycle fatigue testing (periodic dynamitic alternating load stress/strain is applied to CC samples) [66–72], and the other is static-fatigue measurement (a constant force is exerted on the CC samples) [73,74]. High-cycle fatigue research is concerned with the applied maximum stress or strain, i.e. ${\sigma }_{\max }$ or ${\varepsilon }_{\max }$, stress (or strain) ratio *R*, and fatigue number of the cycles ${N}_f$ to reach mechanical (structure broken) or electromechanical failure (critical current degradation). Static-fatigue investigation emphasizes the relationship between the applied stress level and static-fatigue lifetime, i.e. elapsed time to reach a criterion of the retention of critical current.

The first fatigue analysis on CCs was done by Mbaruku *et al.* [66]. The CC made by SuperPower with 50 μm thick substrate and 20 μm thick Cu stabilizer showed no degradation of critical current after 200k cycles under a ${\varepsilon }_{\max }$ of 0.367% and *R* of 0.5. For higher ${\varepsilon }_{\max }$, the degradation of *I*_c_ was observed with lower ${N}_f$. Similar fatigue behaviors can also be found in other studies with stress control models [67,68,71]. Moreover, in those reported by Sugano *et al.* [67], Shin *et al.* [68] and Chen *et al.* [71], both the mechanical and electromechanical ${N}_f$ increased with elevated *R* for the same ${\varepsilon }_{\max }$ or ${\sigma }_{\max }$, indicating that the fatigue strain (or stress) range was a more important factor in determining the mechanical and electromechanical ${N}_f$ than the ${\varepsilon }_{\max }$ or ${\sigma }_{\max }$. Since the metal layer accounts for the main part of CCs, and current-carrying capacity depends on the superconducting layer, thus mechanical fatigue properties are mainly determined by the ductile metal substrate, and electromechanical fatigue properties predominantly depend on the brittle ceramic REBCO layer. Fractographic results showed that mechanical failure correlated with the fatigue cracks in the Hastelloy substrate [67], and the degradation of *I*_c_ was caused by crack propagation in the REBCO layer. The cracks formed and localized in weak areas and in defects that were induced by a slitting process at the CC edge [66,69,70,72], i.e. a full-width CC tape was cut into small-width CC tapes. These cracks grew faster due to higher ${\varepsilon }_{\max }$or ${\sigma }_{\max }$, and as a result, a full-width CC usually had a bigger fatigue tolerance than the cut one [72].

With regard to the static fatigue behavior, there are a few studies on it. De Leon *et al.* [73] reported that the CC had no degradation with an applied stress level of 90% of yield stress ${\sigma }_{\rm{y}}$and elapsed time over 100 hours, whereas *I*_c_ dropped significantly when high stress levels were applied, especially when the applied stress was near or equal to the ${\sigma }_{\rm{y}}$. In addition, their recent work [74] found that for a CC sample simultaneously subjected to axial tension and bending strain in a static fatigue experiment, the bending diameter influenced the allowable applied stress level and the static fatigue lifetime, i.e. for a smaller bending diameter with the same static fatigue lifetime, the applied stress level must be reduced.

#### Shear strength of REBCO CCs

**Figure 6.**
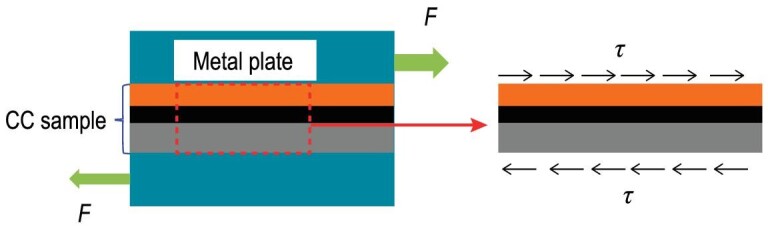
Schematic diagram for measuring shear strength of REBCO CCs.

**Figure 7.**
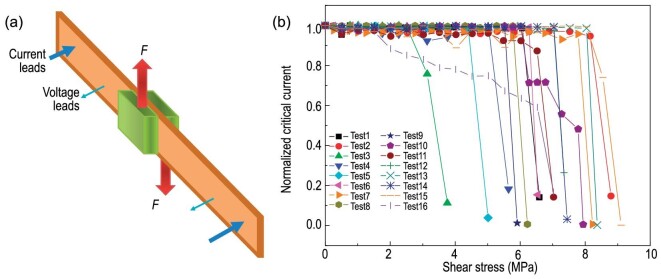
The degradation of critical current under shear stress. (a) Schematic of the shearing experiment. (b) The results of dependence of normalized critical current on the shear stress.

For epoxy impregnated magnet coils made from REBCO CCs, the hoop stress caused by electromagnetic force is a function of the radius. Shear stresses exist at the interface between the epoxy and CC tapes. Hence the shear strength is one of the important mechanical properties of YBCO CCs. As shown in Fig. 6, the shear stress is applied to the CC sample through a pair of soldered metal plates that are under tensile forces. Some researchers measured the shear strength of YBCO CCs. For example, Gao *et al*. proposed a new method, which can realize the pure shear load on the tapes by eliminating the torque effect, to measure the shear strength of YBCO CCs [75]. Liu *et al*. designed an experimental device for applying shear stress along the width direction of the tape [76]. The delamination test of the YBCO tape under shear stress at both room temperature and liquid nitrogen temperature has been performed. In the work by Liu *et al*. [76], it is reported that the shear strength of YBCO CCs is <10 MPa, and the average shear strengths at liquid nitrogen are higher than those at room temperature. Also, as shown in Fig. 7, the dependence of *Ic* on the shear stress of 16 samples was measured. One can see that: (i) all shear strengths are <10 MPa; (ii) for most of the samples, their critical current decreases rapidly, as shear stress is close to the maximum values.

### Electromechanical properties of REBCO CCs in the transverse direction

#### Experimental approaches for transverse electromechanical investigation

**Figure 8.**
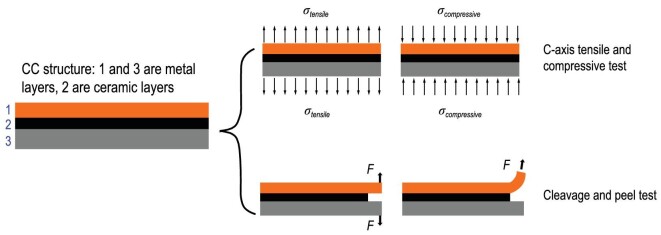
Methods for determining the mechanical strength of the laminar structure of YBCO CCs in a transverse direction.

For a 2G HTS CC, the brittle ceramic layers including YBCO layer and buffer layer are together sandwiched by the ductile metal layers, i.e. silver, copper and substrate layers. For the reason that the fracture toughness of the metal layer, in the order of kJ/m^2^, is several orders higher than the ceramic layer, typically in the order of J/m^2^, the whole CC sample can be regarded as a mechanical structure that the metal layers are joining together by this adhesion [77]. As a result, the bonding strength in the transverse direction is mainly determined by the cohesive bonding strength in the matrix of each ceramic layer itself, and adhesive strength between the interfaces. In order to achieve a better understanding of the mechanical properties of this laminar structure, especially the stress response along the transverse direction, the testing methods are classified into four types based on the exerted stress condition [77], as shown in Fig. 8.

#### Delamination behavior of REBCO CCs

**Figure 9.**
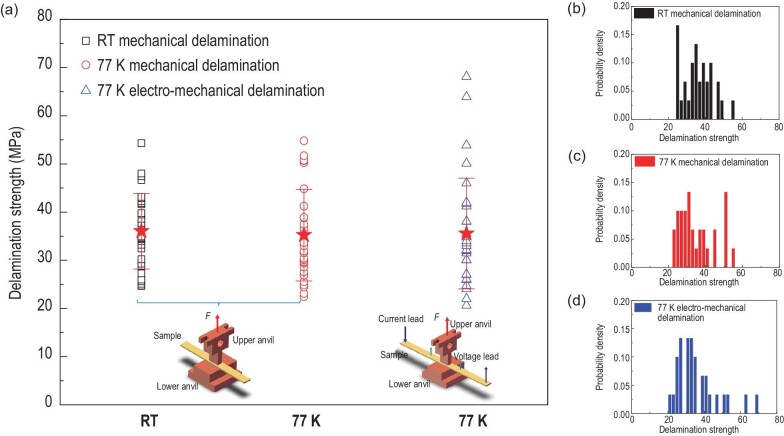
Delamination strength of YBCO CCs from SuperPower Inc., reproduced from ref. [92]. The anvil has a width of 4 mm and a length of 8 mm. The width of the CC sample is 6 mm. The sample capacity of each group is 30. (a) Mechanical delamination strengths of YBCO CCs at room temperature and 77 K along with results of the electromechanical delamination strength. The two inserts are schematics for mechanical and electromechanical tests. (b), (c) and (d) are the frequency distributions of the mechanical delamination strength of YBCO CCs at room temperature, 77 K, and electromechanical delamination strength, respectively. In each group test, 30 samples were continually cut and used for testing. The mechanical delamination strength ranges from 22.5 MPa to 54.8 MPa with an average value of 35.3 MPa at 77 K, and from 24.7 MPa to 54.3 MPa with an average value of 36.0 MPa at room temperature. The electromechanical delamination strength has a maximum of 68.1 MPa, minimum of 20.6 MPa, and mean value of 35.5 MPa. It can be observed that all the experimental results share discrete characteristics.

The transverse tensile test includes anvil [78–92], pin-pull [93] and three-point bending tension tests [94]. In the anvil method, the CC is soldered between a pair of upper and lower anvils, thus the tensile stress is applied through the anvils, perpendicular to the CC surface. This method was first adopted by Van der Laan *et al.* [78] to evaluate the transverse mechanical delamination strength (MDS) of YBCO CCs. Shin *et al.* [82] gave the definition of mechanical delamination and electromechanical delamination, and conducted a series of systematic studies on factors that influence the anvil measurements. For mechanical delamination, physical separation takes place within a layer or between any adjacent layers. The delamination strength is related to adhesion strength. For electromechanical delamination, electrical disconnection happens when the superconducting layer is broken, which is detected by degradation of critical current. For the mechanical delamination test, it is found that the slitting process in fabrication could reduce the result of the MDS due to the crack formation of the superconducting layer near the cut edge of the CC samples [78,80,81,87,88,91]. It was found that the anvil measurement is not influenced by certain factors, like CC sample arrangement between the anvils [86,91], loading speed [84] and thickness of the silver layer [92], and several numerical analyses were conducted to evaluate stress distribution [95] and failure behavior [96] in the anvil test. Nevertheless, all the measured data show the behavior of discrete distribution [79,80,82,84–92,97]. The dispersion degree depends on the anvil size [80,82,87] and the position of the CC with a cut edge [80,86,88]. Figure 9 displays the experimental results of CC mechanical delamination strengths for room temperature and 77 K, as well as electromechanical delamination strength.

**Figure 10.**
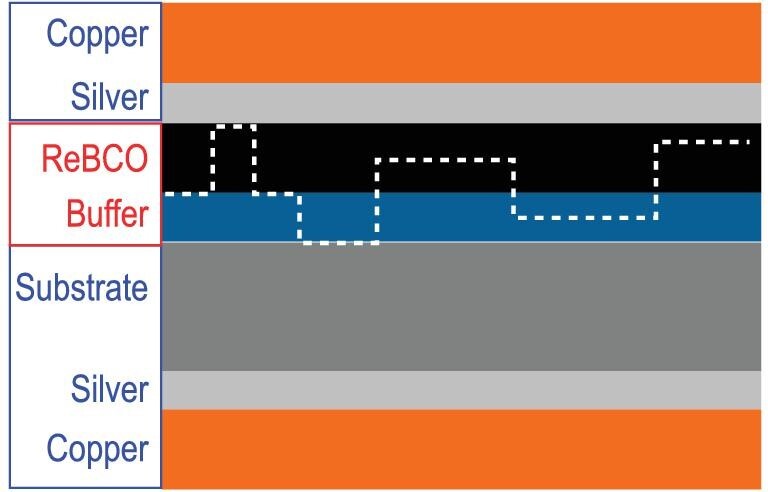
A schematic of a zigzag crack path during the delamination.

The micro analyses [82,86,93], through scanning electron microscopy (SEM), energy disperse spectroscopy (EDS) or optical microscopy, reveal that the delamination sites occur in the forms of both intralaminar fracture of the ceramic matrix and interlaminar cracking at the interface, as shown in Fig. 10.

**Figure 11.**
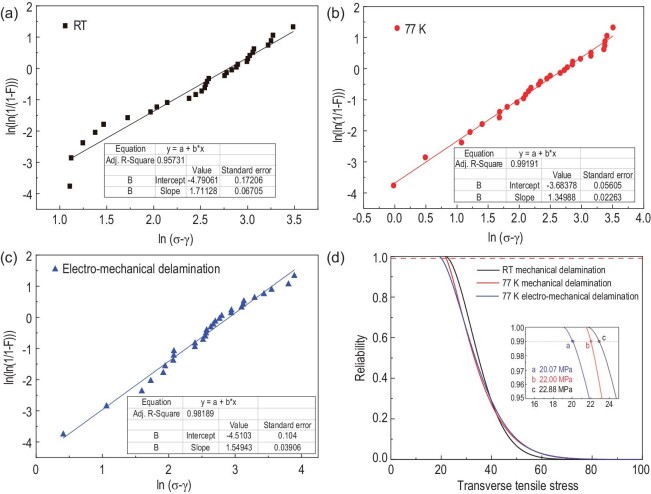
The three-parameter Weibull distributions of the delamination strength of YBCO CCs in different situations, reproduced from ref. [92]. (a) Mechanical delamination at room temperature. (b) Mechanical delamination at 77 K. (c) Electromechanical delamination at 77 K. (d) Weibull reliability distribution versus transverse tensile stress. The distribution shows the reliability of the safety of samples under the transverse tensile stress. Points a, b and c are the corresponding delamination strengths at a reliability of 99%. The corresponding mechanical strengths at room temperature and 77 K are 20.07 MPa and 22.00 MPa respectively. The electromechanical delamination strength is 22.88 MPa at 77 K.

Hence the brittle fractures make the discrete data hard to evaluate just using the average and variance. For this reason, a Weibull distribution analysis is employed to carry out an efficient analysis [82,84,88,97]. In the work of Zhang *et al.* [97], a three-parameter Weibull distribution function [98] was used and a criterion based on the Weibull reliability function was provided. In the Weibull statistical analysis, a Weibull failure function was determined and properly described the data distribution. Weibull reliability distribution as a function of transverse tensile stress can be obtained, from which the corresponding mechanical and electromechanical delamination strengths are determined by a reliability criterion and can be used as a reference for the engineering test and design, as displayed in Fig. 11. After that, the minimum sample capacity and optimum anvil size for proper Weibull distribution statistics were experimentally determined for a standardized anvil test [99].

#### Compressive test for REBCO CCs

The compressive test is opposite to the tension, but it is also implemented by the anvils with compressive stress along the direction that is perpendicular to the tape surface. The early ion beam assisted deposition-pulsed laser deposition (IBAD-PLD) YBCO CCs with Inconel substrate could sustain up to 100 MPa compressive stress with degradation of critical current density *J_c_* <5%, and there was <2% of degradation in *J_c_* after 2000 fatigue cycles [100]. Both the *J_c_* of rolling aided biaxial textured substrate-metallic organic decomposition (RABiTS-MOD) and IBAD-MOCVD YBCO CCs exhibited no degradation under the compressive stress of 150 MPa and 20000 cycles, and this behavior is independent of stabilizer thickness [101]. For IBAD-MOCVD and ion beam assisted deposition-reactive co-evaporation by deposition and reaction (IBAD-RCE-DR) GdBCO (GdBa_2_Cu_3_O_7_-x) CCs, the influence of compressive stress was still negligible with stress up to 586 MPa [102].

#### Cleavage and peeling strengths of REBCO CCs

To evaluate the interface strength of REBCO CCs from the point of view of fracture energy, cleavage [103–106] and peeling [107–109] methods were adopted. Cleavage is defined as the stress occurring when forces at one end of a rigid bonded assembly act to pry the adherends apart. In the cleavage test, the CC sample with a pre-crack that was made at the YBCO/buffer interface is soldered between a pair of double cantilevers. By applying tensile loading at the end containing the pre-crack, model-I-type fracture propagation takes place so that the energy release rate *G* can be measured. *G* at room temperature for YBCO and Ag/YBCO interfaces was measured as 7–10 J/m^2^ and 80–120 J/m^2^, respectively [103,105,106]. Peeling is similar to the cleavage, but the difference is that the CC sample is soldered on one beam, and a peeling force at a fixed angle is applied to the partially peeled arm of the CC sample. The peeling force per unit width was measured with good reproducibility and accuracy [107,109], from which the fracture energy release rate *G* can be derived. Nevertheless, it should be noted that peeling results strongly depend on the peeling angle and the energy contribution from macroscopic plastic deformation at the peeling point, e.g. the results rely on the thickness of the copper stabilizer [108]. Duan *et al.* numerically investigated the effect of substrate thickness on the interfacial adhesive strength of 2G HTS tape by peel test modeling, in which the thermal residual stresses were an important reason for reducing the peeling strength [110].

## MAGNETO-MECHANICAL COUPLING IN THE DEVELOPMENT OF HIGH-FIELD MAGNETS

In 2000, Johansen presented an insightful view on the development of HTS materials, i.e. the mechanical response of these materials in high magnetic fields may be more important than their critical current density [111]. To date, the development of high-field magnets still faces many scientific challenges, such as: (i) under extreme environments (low temperature, large current and high magnetic field), the high-field magnet is subjected to huge electromagnetic forces and stresses, which directly act on the REBCO CC and may lead to degradation of the critical current, delamination, damage and even failure of the superconducting layers. (ii) Compared to a magnet made by typical LTS wires with many twisted multi-filaments, a magnet made by monofilament tape-shaped REBCO CCs show significant screening-current effects. There is remarkable coupling between the screening-current effect and mechanical stress, which was considered as one of the possible reasons for the magnet not meeting expectations. More and more researchers pay attention to the relationship between screening-current effect and mechanical characteristics at present [112–116]. (iii) Because of the strongly non-linear electromagnetic constitutive relations of superconductors, the numerical simulations of magneto-mechanical coupling in the magnet are limited by the huge computation cost and convergence problem. It is imperative to establish an effective multi-field coupling and multi-scale numerical model, which can enhance the development of high-field magnets. In this part, we mainly focus on the mechanical behaviors that are related to the screening-current effect.

### Stress amplification produced by the screening current

**Figure 12.**
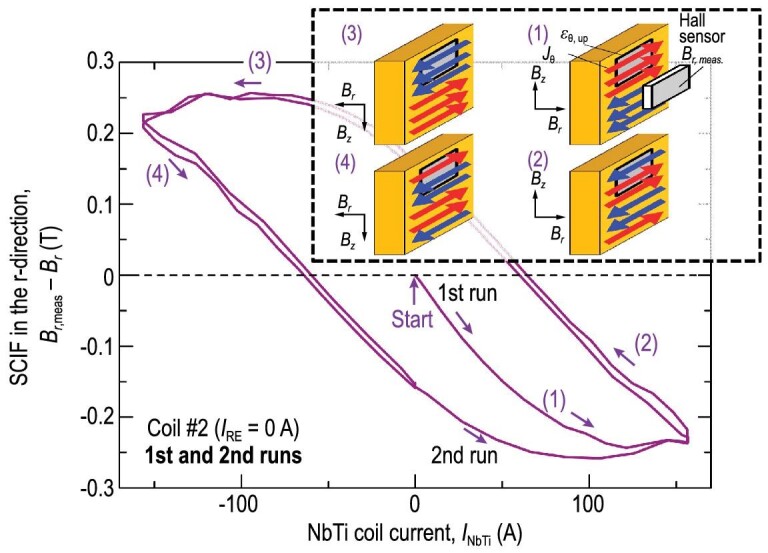
The screening-current-induced field is calculated in the single pancake coil. The inset is the screening-current distribution in the REBCO CC. Reproduced from ref. [121].

In the initial charging state of a superconducting magnet, screening currents are generated as a response to the change of magnetic field and produce an undesirable field in the magnet center [22,117–120]. As illustrated in Fig. 12, the screening currents lead to a considerable reduction of magnetic field intensity, since part of the screening current is opposite to the transport current within the current ramping-up process [121]. The field reduction generated by the screening current is related to the magnetization history of the superconducting coils, which can be described approximately by the well-known Bean critical state model [122,123]. Besides, the decay of the screening current takes quite a long time. Therefore, the screening-current-induced field exhibits the hysteresis effect [121]. In addition to having an effect on the magnitude of the field intensity, the spatially non-homogeous screening current affects the overall target field distribution as well, especially the original field homogeneity along the axial direction.

In comparison with the multi-filamentary wires and tapes, such as NbTi, Nb_3_Sn, Bi2212 and MgB_2_, screening-current effects are reported to be larger in REBCO-CC-based configurations due to the flat and wide surface of commercially available REBCO CCs [124]. The screening-current relaxation can result in a temporal drift of the magnetic field due to the thermally flux creep of a REBCO CC. In a typical insulated REBCO pancake coil, the screening-current-induced field slowly decays over time, and the decay rate can be increased at a higher transport current [125]. Furthermore, the time stability of the field can be more complicated for no-insulation and metal-insulation coils due to the field-charging delay [114]. In general, the field disturbance and temporal drift issue are undesirable and detrimental to the performance of REBCO coils and their applications in NMR, MRI and accelerator magnets, which indeed require high spatial homogeneity and field stability. Therefore, feasible techniques have been developed to eliminate the screening-current-induced field.

With the continuous progress of superconducting magnet technology, the magnitude of the strong central field was greatly improved. Although screening-current-induced field effects have been mentioned since the birth of LTS magnets in the 1960s [22], it is only in the last few years that the high stress level arising from the interaction of the strong field and screening currents has attracted researchers’ attention. Stress modification due to the screening current became one of the most significant technical challenges for the further development of high-field REBCO magnets [126]. Most of the available high-field REBCO magnets are made by dry-winding technology. For instance, an insulated dry-winding REBCO magnet is inserted in the National High Magnetic Field Laboratory (NHMFL) 32 T all-superconducting magnet [127], and a no-insulation dry-winding REBCO magnet is inserted in the NHMFL 45.5 T hybrid magnet [128]. Under the action of complicated loads such as thermal stress and electromagnetic force, the HTS coil undergoes structural deformation. Due to the dry-wound characteristic of the REBCO coil, relative displacement may occur between the contact surfaces, resulting in turn-to-turn separation behavior [129]. After considering the uneven distribution of screening current and magnetic field in superconducting magnets, the analytical solution based on the assumption of plane stress cannot effectively evaluate the mechanical behavior of high-field REBCO magnets [130], and therefore it is necessary to resort to numerical simulations.

In order to reveal the influence of a screening current in high-field REBCO magnets, Xia *et al*. developed a discrete contact model to simulate the screening-current-induced stress-strain in stacked dry-wound REBCO coils [130]. Based on the combination of a discrete contact model and the screening current obtained by the **H** formulation and homogenization method, Xia *et al*. numerically evaluated the local overlarge stress and the non-uniform strain resulting from the screening-current effect [130]. Moreover, the non-uniform radial and hoop deformation along the tape width direction can be observed whilst considering the screening current, and maximum local hoop stresses of the coils are significantly underestimated compared to the case where the screening current is neglected. Li *et al*. extended the discrete contact model to the mechanical behavior analysis of the 18.8 T REBCO magnet, and further explored the effect of friction between adjacent pancake coils [131]. Meanwhile, in contrast to the case where the screening current is not taken into account, a high tensile hoop stress is induced by the screening current in the HTS coils due to the concentration of outward Lorentz force. Thus, the maximum hoop strain and stress of the HTS coils in high-field magnets can be enhanced remarkably by the screening current. Furthermore, the numerical results of Xia *et al*. [130] and Li *et al*. [131] also indicated that the large screening current generated in the magnetization process of REBCO CCs may induce a risk of overstress, posing a threat to the mechanical strength of the high-field magnet. Recently, the screening-current-induced non-uniform strain was experimentally validated by Takahashi *et al*. [121], Yan *et al*. [132] and Li *et al*. [112]. Kolb *et al*. [133] computed the screening-current-induced strain of the prototype REBCO coil. They also compared the calculated results with the observation and post-mortem analysis of the test coil [133]. Both the numerical and measurement results revealed the local degradation and plastic deformation of the REBCO magnet induced by the screening current.

In addition to the high electromagnetic stress caused by the screening-current effect, the winding and cooling stresses during the coil preparation and cooling stages are also significantly correlated with the mechanical and electromagnetic properties of the high-field REBCO magnet. To obtain the winding pre-stress of the coil, a simple 1D analytic solution has been derived based on the combined homogeneous cylinder method (CHCM) [129,135]. Meanwhile, a 2D finite element method based on element birth and death technology was also developed to calculate the winding pre-stress [134,135]. It was found that the stress distributions estimated by the two methods were in good agreement. Recently, a 3D finite element model was built to calculate the contact stress distribution among the turns during the winding process [136,137]. The numerical results indicated that the contact stress along the width of the conductor had non-uniform distribution. It is difficult to measure the winding pre-stress in experiments, and these models still need to be verified in the future. Moreover, the binding force generated by overbanding radial build is beneficial to HTS coils [138,139]. A parametric study on overband radial build has been performed for a REBCO 800 MHz insert of a 1.3 GHz LTS/HTS NMR magnet, and it has been demonstrated that overbanding coils is necessary to keep a small mechanical deformation [140]. Recently, a multi-step analysis was constructed to simulate the accumulated stress distribution after winding, cooling and electromagnetic excitation of REBCO insert coils in a NHMFL 32 T magnet [131]. In the numerical model, the 15 T REBCO insert magnet contains ∼20000 turns of tape. The electromagnetic field computation is based on a combination of the T-A formulation and homogenization technique, and the governing equations can be described as


**Figure 13.**
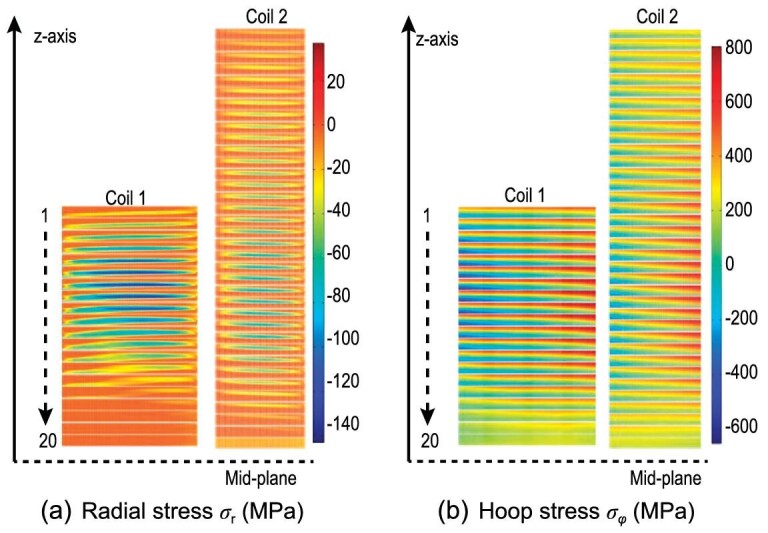
Distributions of (a) radial stress and (b) hoop stress at the fully charged state of the REBCO insert coils of a 32 T magnet. Reproduced from ref. [134].

(3) \begin{eqnarray*} {\nabla }^2{{\bf A}} = - \mu {{\bf J}},\quad{\rm{ }}\nabla \times \rho \nabla \times {{\bf T}} = - \frac{{\partial {{\bf B}}}}{{\partial t}}, \end{eqnarray*}

where the current vector potential ${{\bf T}}$ is only defined in the superconducting conductor, and the magnetic vector potential ${{\bf A}}$ is defined in all the computational domains. The simulation results seen in Fig. 13 indicate that the screening-current-induced hoop stress is higher, which possibly induces local degradations of the current-carrying capacity.

A large number of experiments and numerical analyses demonstrates that the degradation of the current-carrying performance of HTS coils may occur during the cooling and charging stages. Recently, researchers noted that the separated analyses of the electromagnetic-mechanical field give an overestimation of the screening-current effects of high-field REBCO magnets, including screening-current-induced field and stresses [18]. The structural deformation also leads to misalignment between the deformed tape surface and the coil's axis. Therefore, the magnetic field orientation, with respect to the deformed tape surface, and the screening currents change in the excitation process. In addition, it is possible for the screening-current-induced stress and strain to induce the degradation of superconducting properties. Therefore, a few researchers built the coupled electromagnetic-mechanical model to replace the separated model [113,115,116]. Kolb *et al*. studied the effects of the tilting angle and the strain-dependent critical-current relationship on the electromagnetic field of HTS magnets [116]. The numerical results indicated that the distribution of the current density is closely related to the mechanical deformation of magnets. Meanwhile, Yan *et al*. also simulated the magnetization of HTS coils based on the coupling of electromagnetic and mechanical responses [115]. It can be found that the results of the discrete contact model with coupling mechanical deformation are in agreement with the experimental results.

On this basis, a 3D electromagnetic and mechanical coupling model was also developed to study the screening current and the electromagnetic-mechanical behaviors of the coil [113]. Although the electromagnetic field of the coil was still calculated by the T-A formulation, the governing equation for the T formulation needs to be revised by considering the deformed angle, which can be expressed as


(4) \begin{eqnarray*} &&\!\!\!\! {\left[ {\frac{{\partial {E}_z}}{{\partial y}} - \frac{{\partial {E}_y}}{{\partial z}}{\rm{ }}\frac{{\partial {E}_x}}{{\partial z}} - \frac{{\partial {E}_z}}{{\partial x}}{\rm{ }}\frac{{\partial {E}_y}}{{\partial x}} - \frac{{\partial {E}_x}}{{\partial y}}} \right]}^{\rm{T}}\centerdot {{\bf n}}\\ &&= \frac{{\partial {B}_\parallel }}{{\partial t}}\sin \alpha - \frac{{\partial {B}_ \bot }}{{\partial t}}\cos \alpha \\ &&\quad+\,\,\frac{{\partial \alpha }}{{\partial t}}\left( {{B}_ \bot \sin \alpha + {B}_\parallel \cos \alpha } \right), \end{eqnarray*}

where ${B}_\parallel $ and ${B}_ \bot $ are the parallel and perpendicular magnetic field components of the conductor, respectively. The deformed angle $\alpha $ is defined as ${{\partial u} / {\partial z}}$. The resistivity of the REBCO conductor is modified by considering the strain-dependent normalized critical current and n-value, which can be given as


(5) \begin{eqnarray*} \rho = \frac{{{E}_c}}{{{J}_c\left( {{B}_\parallel ,{\rm{ }}{B}_ \bot ,{\rm{ }}\varepsilon } \right)}}{\left| {\frac{{\left| {{\bf J}} \right|}}{{{J}_c\left( {{B}_\parallel ,{\rm{ }}{B}_ \bot ,{\rm{ }}\varepsilon } \right)}}} \right|}^{n\left( \varepsilon \right)}. \end{eqnarray*}

**Figure 14.**
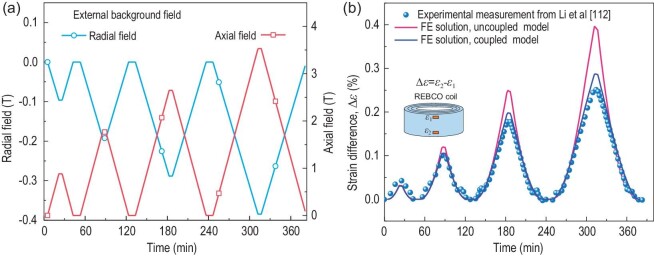
(a) Time evolutions of the external radial field and axial field of a REBCO coil with three turns. (b) Experimental and simulated results of the strain difference between the bottom and upper parts of the outermost layer [113]. The time evolutions of both numerical results are in qualitative agreement with the measured ones [112], especially in low external field conditions. The results predicted by the uncoupled model are significantly higher than the coupled model. Reproduced from ref. [113].

Figure 14 indicates that the mutual interaction between the electromagnetic field and mechanical deformation affects the accuracy of numerical simulations of the electromechanical characteristics.

The structure feature of REBCO CCs suggests a fundamental method for eliminating their screening current, which is to divide the wide superconducting layer into a number of sections, i.e. a multi-filamentary REBCO CC [141]. Yanagisawa *et al*. performed the exploration of multi-filamentary tape in REBCO coils [142]. It was found that multi-filamentary tape can decrease the screening-current-induced field and mitigate the temporal drift of the magnetic field after coil charge. The width size of a narrow filament was revealed to impact the reduction of a screening-current-induced field [143]. Despite the decrease in screening-current effects, the production of a narrow REBCO filament should take care to avoid causing mechanical damage, which is also one of the drawbacks of this method. The application of a multi-filamentary REBCO CC in an ultra-high-field magnet is, to date, still in progress. Another conventional method to reduce screening-current effects is the current sweep reversal method [22], i.e. increasing the transport current above the target value and then reducing to the target. In contrast to the direct ramping-up path, the current sweep reversal method is able to generate a flux barrier and thus mitigates the field drift issue [144]. Better field stability of the REBCO coils after charging can be achieved by increasing the overshooting current of the current sweep reversal method. However, an increased transport current can significantly increase the mechanical stress in a high field as well. Wulff *et al*. [122] stated that the combination of multi-filamentary tape and current sweep reversal method is expected to be a feasible way to decrease the screening current with higher efficiency.

### The thermal stability and mechanical behaviors of HTS no-insulation magnets

Since HTS conductors were discovered, quench protection has always been the main constraint for the HTS magnet due to the low normal zone propagation velocity of the REBCO CC compared to low-temperature superconductors [145–148]. In the past, a superconducting coil was wound with insulation layers, which had relatively low transverse and longitudinal normal zone propagation velocity. Thus, the critical current of the magnet had an obvious degradation during the quench, and the magnet may even have been burned out. Moreover, most of the insulating materials were flexible, which could cause a reduction in mechanical strength in HTS coils, especially when the magnet was subjected to a large electromagnetic force in a high field [149–151]. In 2011, Hahn *et al*. proposed the no-insulation (NI) winding technique and wound a double-pancake (DP) NI REBCO coil [152]. Experimental results showed that the DP coil can withstand more than twice the critical current in an overcurrent test. This means that an HTS coil wound using the NI approach has high thermal stability and self-protection ability.

Many experiments have indicated that burnout of NI coils can be avoided in cases of overcurrent, sudden power failure and heating [149,152–156]. Initially, these phenomena were interpreted as the current bypassing the local hotspot radially through the lower turn-to-turn contact resistance [157]. In 2016, Wang *et al*. numerically analyzed the self-protection mechanism using an equivalent circuit network model [158]. The results showed that except for the local region of the hotspot, the azimuthal current redistributes in the whole coil. As the normal area expands, the azimuthal current and the magnetic field would decrease. Ultimately, the NI coil survives and recovers due to radial shunt. Another advantage of NI coils is defect-irrelevance. Hahn *et al*. experimentally confirmed that the electromagnetic behaviors of a coil containing defects were almost identical to that of a defect-free coil [159]. Thus, this means that the quality of the superconducting tape has a small effect on the electromagnetic property of an NI coil, which will greatly reduce the cost of manufacturing the magnet.

**Figure 15.**
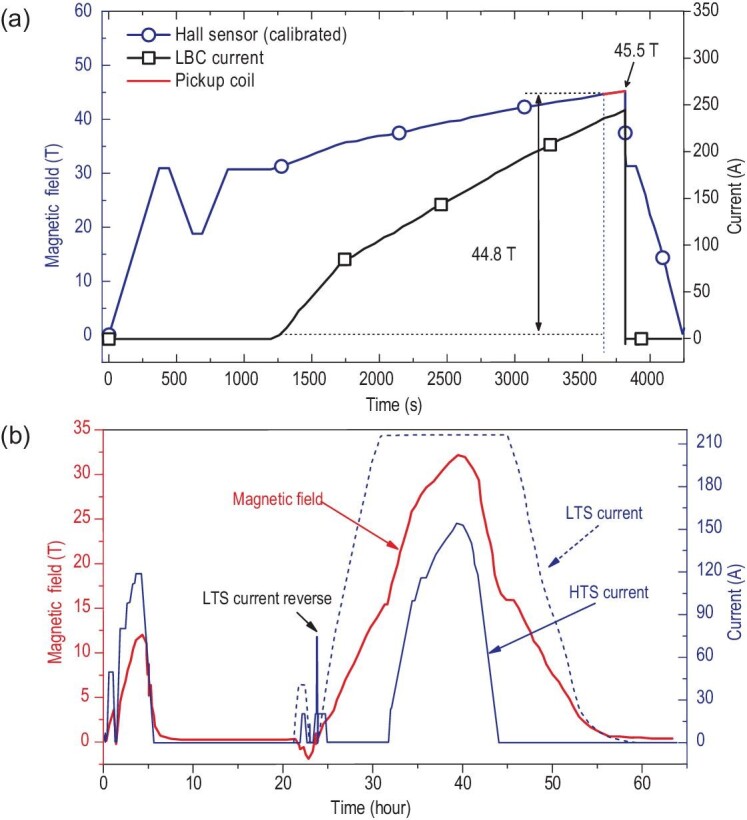
(a) A 45.5 T magnet of the USA. (b) A 32.35 T all-superconducting magnet of China. Reproduced from refs [128,160].

Based on its high current density, thermal stability, mechanical strength and defect-irrelevant behavior, the HTS NI coil has become a candidate for the preparation of high-field magnets [161–163]. In 2016, the South Korean company SuNAM and the Francis Bitter National Magnet Laboratory (FBNML) at the Massachusetts Institute of Technology (MIT) developed a multi-width HTS NI magnet consisting of 26 DP coils, which generated a central magnetic field of 26.4 T [154]. In 2019, the researchers of the National High Magnetic Field Laboratory (NHMFL, USA) inserted a 14.4 T NI magnet into a 31.1 T resistive magnet, and obtained a magnetic field of 45.5 T [128], as shown in Fig. 15(a). In 2020, the Chinese Academy of Sciences (CAS) achieved a 32.35 T magnetic field in an all-superconducting magnet [160], as shown in Fig. 15(b). These achievements indicate that NI technology can potentially be applied in the research and development of ultra-high-field magnets.

However, NI technology also has some disadvantages because of its lower turn-to-turn contact resistance. Experiments and numerical simulations have revealed that NI magnets need more time than insulated magnets to stabilize the magnetic field during charging and discharging processes due to radial shunt [152,164]. In addition, the magnetic field drops to a very low value during a quench and thus the magnet only has a small magnetic field in a lengthy period, which can greatly affect the operational availability of the magnet. In order to decrease the field delay time, researchers have proposed some methods. For example, Choi *et al*. inserted some insulation layers between certain turns of the coil [165,166]. This can decrease the delay time without sacrificing the self-protection ability. On this basis, the metal insulation method, proportion integration control, high resistance layers and smart materials inserted among adjacent turns were also proposed to improve the turn-to-turn contact resistance [167–171]. Although these methods can mitigate the field delay time of an NI magnet, their effects on the mechanical behaviors of the magnet still need to be further studied.

The large electromagnetic force generated by a large current density and high magnetic field often leads to remarkable mechanical deformation and even damage to the magnets. Moreover, thermal stress during a quench also plays a non-negligible role. Lecrevisse *et al*. inserted an NI coil into a resistive magnet [172,173]. There was a lot of induced current generated in the NI coil during a quench, and it was also found that the damage was extended in the coil, and an obvious delamination phenomenon was observed in the tape. The NI magnet inserted in the 45.5 T magnet was also damaged after quenching [128,174], and plastic deformation and cracks appeared at the edge of the conductors in most of the coils. Since magnets operate in a multi-field coupling environment, it is necessary to analyze the electromagnetic-thermal response and mechanical deformation of the NI magnets during a quench.

**Figure 16.**
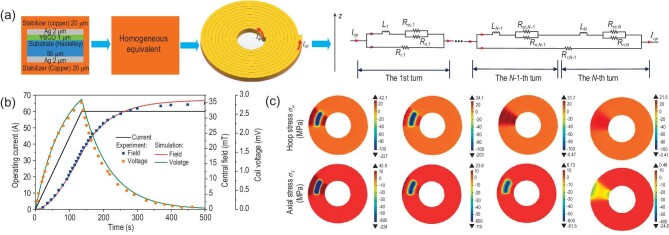
(a) A homogenous model and circuit diagram of an NI pancake coil. (b) The central field and coil voltage from the simulation are in good agreement with those from the experiment. (c) The distributions of the hoop and axial stresses in different surfaces (from left to right: upper and lower surfaces of top pancake coil, upper and lower surfaces of bottom pancake coil) of the double-pancake coil at the end of a heat pulse. Reproduced from refs [175,176].

Based on the homogenous method, a multi-physics quench model was built to study the electromagnetic-thermal behaviors and mechanical response of NI pancake coils during a quench. This involves considering electromagnetic force and thermal stress, as show in Fig. 16(a) and (b) [175,177]. An equivalent circuit model was employed to calculate the distributions of radial and circumferential current in each turn, and each turn was divided into superconducting layers and normal layers. Thus, the current of the superconducting layer of the *m*th turn can be calculated as


(6) \begin{eqnarray*} {E}_c{l}_m{\left( {\frac{{{i}_{sc,m}}}{{{I}_{c,m}}}} \right)}^n - \left( {{i}_m - {i}_{sc,m}} \right){\rho }_n\frac{{{l}_m}}{S} = 0, \end{eqnarray*}

where ${E}_c$ is the critical electrical field. ${i}_m$ and ${i}_{sc,m}$ represent the circumferential current and the current of the superconducting layer in the *m*th turn, respectively. Moreover, with the radial Joule heat generated in the turn-to-turn contact surface, the governing equation of the thermal model was given as


(7) \begin{eqnarray*} dC\frac{{\partial T}}{{\partial t}} &+& \nabla \centerdot \left( { - k\nabla T} \right) = {\rho }_n\,\,\left( T \right)\left( {\frac{{{i}_m - {i}_{sc,m}}}{{{S}_c}}} \right)\\ &&+\,\,{\rho }_r\delta ( {r - {r}_j} )( {\frac{{{j}_m}}{{{S}_k}}} ) + {Q}_{heat} \end{eqnarray*}

where the delta function $\delta ( {r - {r}_j} )$ was introduced to describe the radial Joule heat generated in the turn-to-turn contact surface. Numerical results showed that the stress and strain are mainly affected by temperature in the self field and both of them increase as the temperature increases. For the DP coil, the temperature rise of the coil induced by the heater was also the main influence on the stress distribution in the self field, as shown in Fig. 16(c) [176]. In the high field, the mechanical deformation of the coil was affected by the combined action of the temperature rise and the electromagnetic force.

Although many HTS NI magnets are fabricated by assembling the modules using a pancake-wound method, it is also found that connecting each pancake module with the joint makes it difficult to sustain a persistent current in the magnet [120,178]. Considering the above disadvantage of pancake winding, a layer-wound technique has been developed in recent years, and the conductor was wound on a bore tube. Considering the requirement of a spatially homogeneous magnetic field in an NMR magnet [179], the layer-wound coil is preferred as it can achieve a more homogeneous magnetic field compared to the pancake coil.

Choi *et al.* found that the NI layer-wound coil had a time delay during charging or discharging and a self-protecting feature during a local quench, which is similar to the NI pancake coil [180]. A small NI REBCO layer-wound coil with a self-inductance of 1.62 mH was built and tested by Chiba University in Japan in 2016 [181]. The coil had a charging delay time constant of ∼3000 s at 4.2 K. In the experiment, an excessive increase in the induced current resulted in thermal runaway of the coil. Burnouts of the REBCO CC were found near the copper electrodes, and buckling was also observed for all layers of the layer-wound coil. Moreover, Jiang *et al.* designed and fabricated a small-scale REBCO NI layer-wound coil with an inner diameter of 15 mm and a length of 38 mm [182]. When the coil was tested in a 31.5 T background field, both peeling and buckling appeared in the coil. Thus, the thermal stability and mechanical response of layer-wound coils in a high field need to be further explored for the reliability and stability of the magnet.

A multi-physics quench model has been presented in order to study thermal stability and mechanical behaviors in an NI layer-wound coil [183]. The layer-wound coil had high thermal stability based on radial shunting through the low turn-to-turn contact resistance. The field delay time can be reduced by increasing turn-to-turn contact resistance with the thin metallic cladding technique. The increase of the peak temperature for coils was not significant. The results indicated that it was feasible to reduce the field delay time without sacrificing high thermal stability by designing a layer-wound coil with relatively high contact resistivity. During a quench, the fast temperature rise results in remarkable strain of the coil. It can be expected that a large stress may be induced in the coil if the duration of the heat disturbance is long enough, which could result in permanent damage or degradation.

Recently, a new winding method was proposed to reduce the field delay time of the NI layer-wound REBCO coil, which was termed ‘intra-layer no-insulation (LNI)’ [184]. A polyimide sheet and a copper sheet were both inserted between different layers of the layer-wound coil. The experimental results indicated that not only can an LNI coil reduce the magnetic field delay time, but it also has high thermal stability. In 2021, an LNI REBCO coil connected to an insulated Bi-2223 coil was tested under a background magnetic field of 17.2 T [185]. A central magnetic field of 31.4 T was generated in the magnet and there was no degradation in the REBCO coil after a quench. Li *et al.* investigated and compared the ramping loss and mechanical characteristics of the layer-wound coil and LNI coil via a hybrid numerical model [186]. The LNI winding approach can significantly reduce the ramping loss energy in the whole charging process, and thus an LNI coil has a higher thermal stability margin. Due to the combined action of the cooling process and Lorentz force, the copper sheet of the LNI coil experienced relatively high stress, while the magnitude of stress generated in the REBCO CC of the LNI coil was almost the same as that of the layer-wound coil. This means that the inserted materials have a negligible effect on the mechanical characteristics of the LNI coil.

Some numerical simulation methods have been developed to calculate the screen current distribution and mechanical characteristics of NI coils [187–190]. For example, a 2D axially symmetric model based on the H-formulation was proposed in order to estimate the electromagnetic properties of NI coils [187]. Not only can the method be used to simulate the screening current, but it can also directly capture the distributions of the axial and radial current density. Recently, the method was also applied to study the screening-current-induced mechanical response of the NI coil during the charging process [188]. Moreover, it is assumed that the conductor can be divided into several filaments along the width direction. Two refined circuit models have been built to analyze the screen current distribution of NI coils [189,190]. These methods will also help to achieve a detailed mechanical analysis of an NI magnet induced by the screen current in the future.

The effect of mechanical strain in high-field magnets is significant, and there are still many unresolved key mechanical issues. For example, as the coil is deformed, both the turn-to-turn contact resistance and thermal resistance will change, but these factors are not taken into account in the current numerical analysis. Furthermore, the mechanisms of the bulking, delamination and fatigue of the magnet, observed in the experiments, need to be clarified quantitatively and accurately by numerical simulations, including the screening current effects during the quench process. This is important for the development of ultra-high-field magnets. The simulation results on the stress or strain during the quench are reasonable. The variation trend of mechanical deformation is similar to the temperature in the self field, which means that the strain can be used to determine the quench in the magnet. The quench detection method based on the measurement of strain has also been used in LTS [191]. Namely, relevant experiments should be developed to validate the mechanical simulation results. Meanwhile, a reliable real-time monitoring and testing method for stress or strain needs to be explored in high-field magnets. Therefore, in order to ensure the stable and safe operation of HTS magnets, it is necessary to develop refined electric-magnetic-thermo-mechanical coupling models so as to understand the mechanism underlying the mechanical failure of the magnets. Furthermore, an effective quench detection method should also be explored in order to achieve the quench protection of HTS magnets.

## CONCLUSION

With a further increase in the magnetic field strength of superconducting magnets, it can be predicted that the influence of mechanical effects related to their electromagnetic properties will be significantly enhanced. The authors think that in the future it will be necessary to further focus on research from the following aspects. For REBCO CCs, (i) the low shear strength should be enhanced to improve the interlaminated properties. (ii) The fatigue problem of the superconducting tape with current-carrying under cyclic loading should be given priority attention. For high-field magnets, (i) the superconducting magnets can be optimized based on the results of multi-field simulation to enhance the reliability and stability of magnets. Thus, calculation methods with high efficiency and precision should be used to overcome the requirement of too much computation caused by the 3D refined electromagnetic and mechanical models. (ii) Some structures should be developed in order to achieve the effective stress management of the superconducting magnets in a high field, to reduce strain-induced degradation. (iii) Currently, it is still too costly to build high-field superconducting magnets. To maximize their application, the total cost should be reduced in the future. In addition, the development of high-field superconducting magnets needs the participation of more and more researchers majoring in mechanics.
